# A Cohort Model and High Impact Practices in Undergraduate Public Health Education

**DOI:** 10.3389/fpubh.2019.00132

**Published:** 2019-05-29

**Authors:** Karin Joann Opacich

**Affiliations:** School of Public Health, University of Illinois at Chicago, Chicago, IL, United States

**Keywords:** learning communities, cohort model, undergradaute curriculum, public health education, high impact practices

## Abstract

Developing curriculum that is more than a collection of courses necessitates articulating philosophy and principles that undergird curricular decisions. While faculty are accustomed to expressing ideas within their realms of content expertise, building consensus around educational philosophy and pedagogy may be less common but equally important to assure coherent curriculum. Such discussions lead to intentional curriculum. When attuned to intent and combined with high impact practices, curriculum is likelier to result in student success and engagement. Since public health by nature entails community interaction, opportunities to think and work in a variety of communities reflects the work in the public health arena. Building a community of learners in the context of a highly diverse urban campus requires very deliberate curriculum planning and design. The likelihood that learning communities will emerge spontaneously is reduced when only a small proportion of students live on or near campus, and most spend considerable time commuting. Virtually all undergraduate public health students have responsibilities beyond academics, including employment, family caregiving, religious obligations, etc. Since most undergraduate students in this setting are first generation to higher education, learning communities and other high impact practices become even more important to provide meaningful baccalaureate education. Such communities evolve most efficiently when integrated into the curriculum design. By implementing a cohort model, not only can faculty participate and facilitate the evolution of a community of learners, they can employ other high impact practices designed to enhance and compound public health content and processes. Undergraduate public health students in this setting take all of their core courses (32 semester hours) together in a prescribed sequence. Faculty have clear understanding about what preceded a course and what follows. Every course entails both individual work and group collaboration. Students come to understand each other's strengths and needs, and with rare exception, they support each other on the journey and share some mutual successes. Both expected and unintended outcomes of this approach are conveyed in this article along with a few cautions for those considering these strategies for undergraduate public health education.

## Introduction

The public health enterprise can best be described as an arena to which many different professionals representing a wide array of disciplines and perspectives come to solve health challenges faced by communities and populations. Subsequently the public health enterprise leads to myriad options for educating those who aspire to resolving public health challenges. Even for programs accredited by the Council for Education in Public Health (CEPH), curriculum development requires many choices about what we teach and how we take into consideration industry standards and expectations, institutional missions, faculty expertise, student audiences, and curriculum aspirations. Ultimately, curriculum is an expression of what faculty chooses to profess to assure that learners achieve a set of clearly articulated curricular learner outcomes that exceed the mere acquisition of knowledge.

Developing curriculum that amounts to more than a loose collection of courses necessitates clear communication of philosophy and principles undergirding curricular decisions. While faculty are accustomed to expressing ideas within their realms of content expertise, building consensus around educational philosophy and pedagogy is less common but equally important to assure coherent curriculum. Such discussions lead to *intentional curriculum*. When attuned to educational intent and combined with high impact practices, curriculum is likelier to result in student engagement and success, especially for historically underserved students. Since public health by nature entails community interaction, most often with vulnerable populations, opportunities to prepare students to think critically, collaborate successfully, and solve vexing problems is central to public health education. The cohort model represents one expression of intentional learning communities, a high-impact practice known to be powerful and highly compatible with education and practice in public health.

This paper will address the cohort expression of learning communities that has been implemented in the baccalaureate program at UIC since its inception. It comports with the learning community as described by Gabelnick et al. (1) to be:

*the purposeful restructuring of the curriculum by linking or clustering courses that enroll a common cohort of students. This represents an intentional structuring of the student's time, credit, and learning experiences to build community, and foster more explicit connections among students, faculty, and disciplines (p. 6/7)*.

## Brief History and Evolution of Thought About Learning Communities

According to Barbara Leigh Smith, who spearheaded a project to chronicle the national history of learning communities (2), interest in learning communities advanced in three waves beginning in 1920. Early experimental efforts exemplified by Meiklejohn's approach in the late 1920s at the University of Wisconsin, employed a pedagogy that integrated active learning with theory and practice in the interest of exploring democracy and issues challenging society (3). His experiment, although perceived as having tremendous impact on students, was abandoned by the university after 5 years. The second wave emerged in the 1960's and focused on alternatives to traditional segmented structures in the academy, and these attempts resulted in innovations but also met with resistance. Some of those innovations, (e.g., student-centered learning, writing across the curriculum, active learning, and interdisciplinary programs) managed to survive (2). In the mid-1980s the notion of learning communities was again resurrected, and research emerged attesting to the tremendous value and potential of an array of learning communities. Educational researchers began to document the power of learning communities to engage students and to engender profound learning, sometimes referred to as *deep learning* (4, 5) Wharburton (6) contrasts *surface learning* with *deep learning as, “…paying attention to underlying meaning. It is associated with the use of analytic skills, cross-referencing, imaginative reconstruction and independent thinking.”* In contemporary literature, proponents of deep learning note qualitative differences in learning that share attributes with learning communities. Most studies are qualitative, although a few quantitative studies do numerically measure the power of learning communities. Some literature does specifically point to the compatibility of learning communities with baccalaureate public health education (7, 8).

## Baccalaureate Public Health Programming in the UIC Context^1^

“With the 9th highest overall score on U.S News and World Report's ethnic diversity index, UIC's student body is one of the most diverse in the nation” ^2^Over 30,000 students are enrolled at UIC, most commuting from the surrounding metropolitan area. More than 19,000 of those enrolled are undergraduates with nearly 90 undergraduate majors from which to choose. Although the School of Public Health was established almost half a century ago, baccalaureate programming is a newer phenomenon introduced on this campus in 2012. At present, more than 65% of UIC undergraduate public health students identify with underrepresented racial and ethnic minorities^3^ reflective of even greater diversity than the campus as a whole.

The UIC Bachelor of Arts in Public Health has embraced four theoretical planks in its approach to teaching and learning in public health. The first of these is a commitment to principles of *liberal education*. Liberal Education & America's Promise (LEAP), an initiative launched in 2005 by the American Association of Colleges and Universities (9), has permeated higher education and is deemed to be central to the development of “an educated citizenry,” a major impetus for the undergraduate public health movement (10–13). The second plank, the notion of *confluent education*, first described in 1971 by Brown (14) and since elaborated by others (15, 16), was selected to give voice to student experience bridging a gap between cognitive and affective understandings, particularly since the student body is largely comprised of first generation college learners whose family stories are replete with health challenges and health disparities of concern to public health. The third plank of the educational philosophy is *action learning* about which much has been written (17–20). Students are taught to be agents in the production of their own learning to assure that learning continues well-beyond the classroom experience.

Last and at the crux of this paper, the fourth plank, *community engaged participatory learning*, shares many features with community-based participatory action research (21). Students quickly come to realize that they both contribute to and benefit from the understandings of their cohort members as well as from people in the communities who experience public health challenges. Public health by nature is collaborative, so experience in community and learning in community is highly compatible with public health practice. When students of public health function within a cohort, they experience many of the same phenomena that they will encounter working with and within a variety of communities from vulnerable populations to professional associations (22). Undergraduate public health students at UIC complete 60 semester hours of general education including three public health pre-requisites (nine semester hours fulfilling general education requirements) and a newly added two semester hour *Foundations for Public Health* course in their first 2 years of study after which they matriculate into the upper division major. Students can declare the pre-public health program as incoming first year students, internal transfer students, or external transfer students before matriculating to the full major in the junior year. The upper division major begins once a year in the fall although students may be admitted to the pre-major phase throughout the academic year. Students in the major move through all 13 upper division public health core courses (35 semester hours) as a cohort^4^. They plan their remaining 25 semester hours of selectives^5^ and electives in accordance with their individual interests and pathways. To date, a single cohort has been as large as 43 students, but sub-cohorts will be necessary as the program grows. This particular cohort model can be described as a long-term (2 year), cross-curricular, face-to-face, public health student community that is supplemented with a digital learning platform (Blackboard).

**Figure 1:**
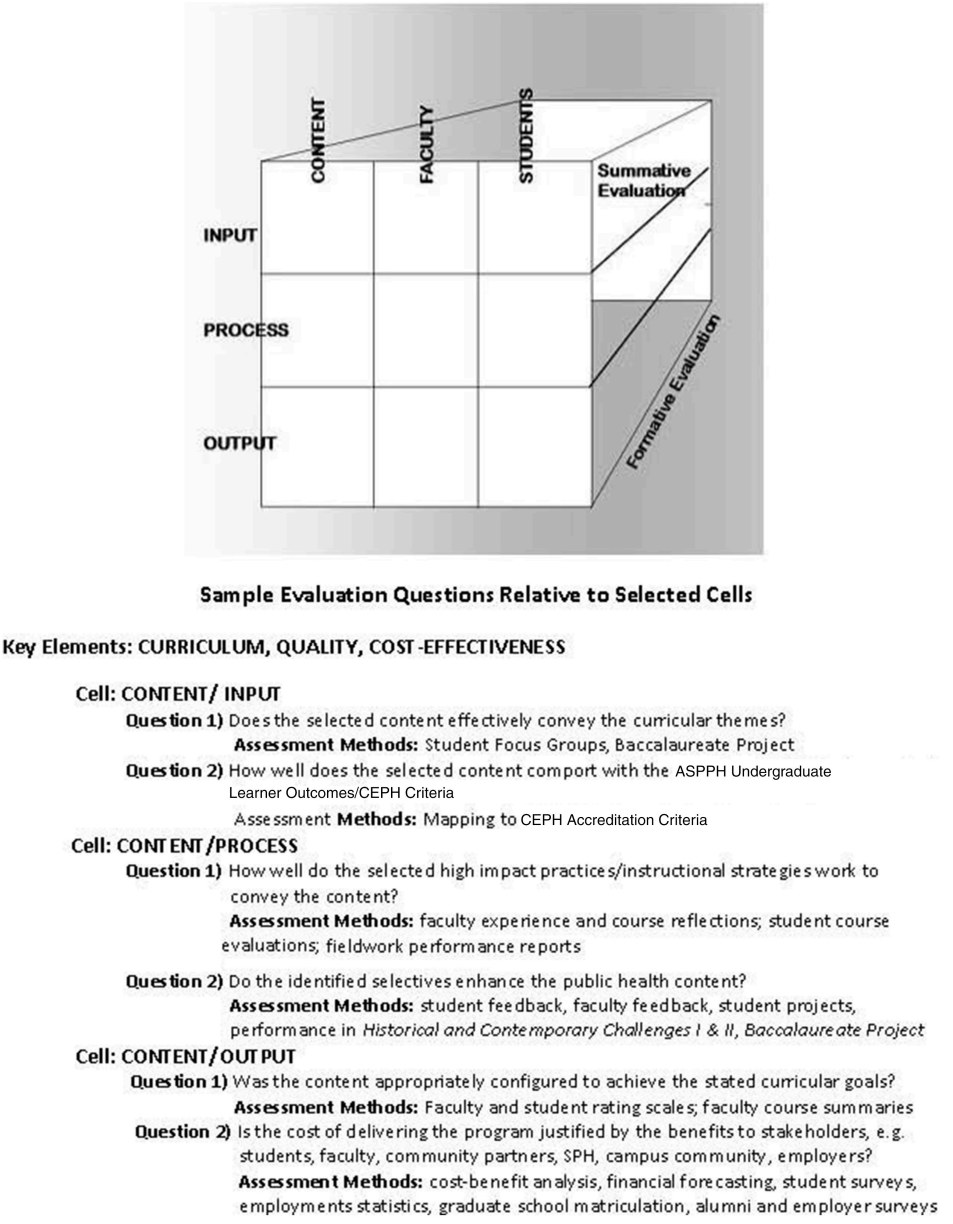
Matrix model of program evaluation.

The overarching curricular themes, the curricular goals, and the curricular learner outcomes appear in Table 1^6^. The program was developed as the Undergraduate Learner Outcomes were emerging in the Framework for the Future initiative^7^. The baccalaureate program is situated in a School of Public Health and was judged by CEPH^8^ to be in full compliance with standards set forth for undergraduate public health programs. Ongoing program evaluation is organized by a fluid Input-Process-Output model predicated on program evaluation concepts introduced in the literature by Stake (23–26). These concepts, adopted in turn by Opacich (27) for application to academic program evaluation ultimately informs our current model of program evaluation that allows for multi-dimensional assessment of all aspects of the program including but not limited to the effectiveness of the cohort model. See Figure 1^9^. Evaluation priorities change from year to year, and a sub-committee of the Undergraduate Oversight Committee determines the focus of assessment crafting new questions, selecting methods, and developing tools as needed.

**Table 1:** UIC baccalaureate in public health-overarching curricular themes, goals, and learning outcomes.

**Overarching Curricular Themes**	
**Health as a Moral Endeavor** *exploring the moral importance of health and healthcare considering individual and societal commitments and obligations including the use of limited resources*	
**Health Equity** *having equitable access and the means and resources to attain one's full life potential*	
**Life Course Perspective** *the cumulative evolutionary, pre-generational, pre-natal, and life events and circumstances that influence health at any one point in time*	
**One Health** [Human-Animal-Environment] *the inextricable relationship among animal, human, and environmental health as determined by, e.g., evolutionary biology, human behavior, and environmental phenomena*	
**Cultural Relevance** *the lens through which life events are experiences and interpreted and through which meaning is ascribed*	
**Local/Global Impact** *appreciation for the global systems that influences the processes, dynamics, and activities of the world's populations; health as a multi-faceted state shaped within, e.g., biological, socio-cultural, geographic, economic, and political contexts*	
**Curricular goal**	**Curriculum learning outcomes**
***Upon completion of the baccalaureate curriculum in public health, graduates will:***	
1. Rise to the challenge of understanding the world in a nuanced way expressing a broad world view and an expansive view of health.	a) Explain the inter-section of human rights and principles of social justice in the production of population health, health equity, and health disparities. b) Analyze historical and contemporary public health events from multiple perspectives. c) Identify and discuss major public health challenges for local, national, and global populations.
2. Be informed, attuned, and energized advocates of health accepting individual responsibility to effect positive change.	a) Discuss the characteristics, limitations, and evolution of health care systems. b) Describe the social, economic, and political processes that influence public health policy and public health services. c) Articulate how human, animal, and environmental health interact and impact the health of populations.
3. Demonstrate skill in critical and analytical thinking.	a) Describe the methods used to measure health status, promote public health, and curtail disease. b) Discriminately apply scientific information and data to public health endeavors. c) Demonstrate the use of selected strategies and tools used for measuring population health.
4. Communicate effectively both orally and in writing with a variety of audiences.	a) Apply critical reasoning to select or develop public health related messages. b) Develop reasoned arguments in support of public health premises. c) Describe culturally appropriate strategies to promote health.
5. Be sensitive and astute observers.	a) Describe socio-cultural, economic, behavioral, and other contextual determinants of individual and population health. b) Explain the importance of cultural practices, values, and perspectives in the assessment and development of public health strategies. c) Discuss the importance of collaboration with professional and non-professional stakeholders in the interest of public health.
6. Commit to being educated consumers of health information.	a) Explain the significance of incorporating perspectives from an array of disciplines to inform public health efforts. b) Access public health information and data using credible resources and information technology. c) Promote public health through presentation of accurate and relevant information.
7. Apply skills and tools acquired to an array of roles in the realm of employment contributing directly or indirectly to public health.	a) Define public health and describe activities in the public health arena. b) Critically assess their own roles and potential contributions to public health in light of their planned career trajectories. c) Explain the importance of developing strategic partnerships to promote public health.

## The UIC Experience: Challenges and Opportunities Associated With the Cohort Model

### Designing, Facilitating, and Nurturing Learning Communities

Designing, facilitating, and nurturing learning communities in any setting takes skill and effort. Building a community of learners in the context of a highly diverse urban campus requires deliberate curriculum planning and design. The likelihood that learning communities will emerge spontaneously is reduced when only a small proportion of students live on or near campus, and most spend considerable time commuting. Today's undergraduate public health students commonly have responsibilities beyond academics, including employment, family caregiving, religious obligations, etc. For first generation college students, learning communities and other high impact practices become even more important to provide meaningful baccalaureate education (28–31). The learning communities evolve most efficiently when integral to curriculum design.

Although proponents of learning communities (32–41) express some variation in characteristics, there is general agreement about the intent and the potential outcomes of true learning communities asserted by Lenning et al. (42) in *Powerful Learning Communities:*

*In a powerful LC, there is optimally effective and ongoing interplay and collaboration among the community's members as they strive for specified common learning goals, and the result is deep learning that is maximally insightful and useful as it pertains to those goals. The members of the group express mutual trust and loyalty, share ideas, and support one another* (p. 9).

Facilitating effective learning communities necessitates specific attention to faculty development as it aligns with chosen models for student learning and compatible instructional strategies.

### Relevant Faculty Development

As is true of any best practice in education, faculty invested in fostering engagement in and beyond the classroom need to be developed and supported. A Master Teacher Mentoring (MTM) program was implemented at the inception of the UIC program, and it aims to acculturate potential faculty to the curriculum as a whole and a single course in particular. Mentees practice instructional strategies that are consistent with the curriculum philosophy under the auspices of faculty who have demonstrated teaching success. Mentees have included advanced public health practitioners, doctoral students, post-doctoral fellows, and academic professionals whose regular scope of work is not teaching. Participants are paired with a master teacher in a course that aligns with their expertise, and a learning contract is negotiated. Additional resources for faculty development are available through the campus.

The literature indicates that there is a modicum of faculty skepticism about the use of cohort models in education, largely because sub-groups can tend to ban together to target faculty or to make unreasonable demands. Conflict of any kind, whether in the classroom, on the campus, or in the community requires insight and skilled management. Public health faculty might reframe these inevitable occurrences as opportunities to teach mediation and negotiation! Issues and ideas are exchanged in faculty meetings and the annual faculty retreat. Most assuredly, considerable attention must be paid to planning learning activities, classroom management, and classroom culture. It is also imperative that faculty collaborate across the curriculum to foster and maintain that culture. When expectations are clear about (e.g., class attendance, preparation for class, active participation in discussions, and contribution to group assignments), it is likelier that students will form the habits that bode well for their individual success as well as success perceived by both teachers and cohort peers. Within the cohort model, faculty can participate and facilitate the evolution of a community of learners using other high impact practices (43) to enhance and compound public health content and processes.

### (re)Shaping Student Learning

Faculty are urged to re-examine their assumptions about college students and teaching/learning. Secondary education continues to be scrutinized and found to be inconsistent in preparing young students for college and beyond. Federal programs such as *Race To the Top*^10^ were intended to incentivize improvements and innovations especially for underperforming school systems often challenged with inadequate resources. Adding to the complexity of the modern college environment, literature has been emerging about the impact of *No Child Left Behind*^11^ on the learning habits and outcomes of a generation of students (44, 45) Alumni of NCLB are now on college campuses, and they are unlikely to mirror the academic experiences of the professoriate. While most students come to college eager to learn, some arrive with less than robust portfolios of academic skills necessary for college success.

In the Chicago metropolitan area, school funding, school violence, and school closures most certainly impact secondary education. The University of Illinois at Chicago (UIC), like many colleges and universities across the country, has implemented student success initiatives aimed largely at supporting first-year students and bolstering their eventual success in achieving a college degree. Intentionally designed to engage and enhance learning LCs can be effective success strategies. However, students accustomed to more traditional surface learning and knowledge transfer approaches may need to be acculturated to a different paradigm of engagement and accountability. The close learning community may very well be an important factor in student persistence and success (46, 47).

Of the high-impact educational practices described by Kuh (43), the UIC baccalaureate curriculum in public health routinely incorporates nine of these: common intellectual experiences; learning communities; writing-intensive courses; collaborative assignments and projects; undergraduate research; diversity/global learning; service learning/community-based learning, internships; and capstone courses and projects. While the literature shows that each alone can add value to the learning experience, the cohort model seems to magnify all the others. When high-impact educational practices are implemented, processed, and distilled in the cohort context, all members benefit by compounding what they might have learned and experienced individually and leading to richer understandings. At this time, midway through the seventh year of operation, 6-year graduation rates for public health hover around 95%, well above the campus average of 6 year graduation rates of 59.7% for first-time, fulltime attendees according to 2017 institutional data^12^ The graduation rate for public health students is no doubt influenced by a 3.0 GPA graduation requirement in the major^13^, a higher standard than most programs on campus, but with rare exception, even students who have been on academic probation or have experienced other challenges have managed to graduate and have been celebrated by their peers. Approximately 20% of UIC baccalaureate public health graduates matriculate to graduate and professional programs immediately, and the remaining 80% of graduates enter the workforce in a variety of public health related settings and jobs. Yet another wave of working graduates seem to be entering graduate school within a few years of degree completion, and more precise data will be forthcoming as alumni surveys are disseminated. Establishing and nurturing learning communities affords both challenges and opportunities. Both expected and unintended outcomes in this highly diverse urban public university are discussed below.

## Discussion

### Advantages of the Cohort Model

Since the program has required students to progress through the core courses together in the same sequence, faculty are well-apprised of where and how their respective courses are situated in the curriculum. They know what learner outcomes students will have been expected to achieve. Unlike courses that may host students at different entry points and widely different levels of understanding and skill, faculty know what students will have achieved so that they can set expectations scaffolding new learning and opportunities accordingly. Faculty carefully communicate and coordinate across the curriculum during the academic year, at the annual faculty retreat, and most certainly when changing any major assignment or component of a course. This practice minimizes unplanned redundancy and renders a clearer picture of how the curriculum unfolds. Attention to priorities, sequencing, and purposeful repetition allows for more focused program evaluation and illuminates needed revisions. When student performance does not meet expectations or when students express a need for further instruction, it is somewhat easier to diagnose where we have fallen short or where we might accommodate their requests in an informed, systematic fashion.

Virtually all classes entail both group assignments and individual assignments, and both the proportion and assessment of group work is informed by the education literature. Students in a closed learning community have the opportunity to collaborate with different sets of peers exploring their particular talents and working toward common goals. The cohort provides a level of familiarity yet allows for both faculty and peer feedback in the execution of assignments. Unlike approaching course assignments in groups of strangers, students are more accountable to each other. Since public health students take 12 of 13 of their core courses (32 semester hours) together in a prescribed sequence, both students and faculty have clear understanding about what preceded and what follows. In both individual work and collaborative efforts, students come to understand each other's strengths and needs, and with rare exception, they support each other on the journey sharing mutual successes and individual achievements. They develop work skills, patterns, and habits that can be applied in the public health arena with the understanding that they can be productive even if they might not like everyone in the group.

Direct feedback during the junior year professional topics seminars, exit surveys, and alumni focus groups have yielded positive feedback about the cohort model. There are always a few students who dislike some of the necessary features, e.g., the schedule, because it does not always comport with individual preferences, but they do not seem to link the scheduling restrictions with the cohort model. It appears that students form a camaraderie that often extends beyond the classroom and curriculum that bolsters them through the life events and stressors that they experience. For the most part, students encourage and support each other, but occasionally, faculty mediate conflicts or intervene when a student is underperforming affecting the cohort. If students have issues with faculty that cannot be satisfactorily resolved, the matter is referred to the Office of Student Affairs for mediation, but these incidents have been rare. Students generally appreciate their ready access to faculty and seek their counsel. Most cohorts have sustained contact with each other through digital media long after graduation, and most keep the program abreast of their jobs and achievements.

### Challenges

In this particular urban setting, students who are navigating higher education for the first time in their family histories seem to express more stress and anxiety than there are resources available to support them. Some of these stressors are related to academic performance, but most pertain to finances^14^, living situations, and family and relationship circumstances beyond the classroom. Professors receive many letters of accommodation from the Disability Resource Center, and in the classroom one can expect students who are diagnosed and those who have yet to be diagnosed with conditions that impact learning and group dynamics. For some, the cohort model provides a peer group that serves as a surrogate family providing safety, acceptance, and some measure of predictability. Occasionally students behave in ways that are disruptive to the community of learners bringing their own histories and issues to the forefront. Managing dissonance requires clarity and cohesion on the part of the faculty and support from administrators. A thorough understanding of campus resources can be very useful in meeting both student and faculty needs.

## Institutional Challenges to Cohort Model/Learning Communities and Other Best Practices in Education

A staunch proponent of learning communities, Smith (2, 48), observes three challenges in traditional university structures that are bettered through the formation of learning communities: the challenge of diversity, the challenge of institutional change, and the challenge of purpose. These are certainly relevant in today's academies and reflective of the challenges at UIC.

### Diversity of Students

Along with diversity comes a range of perspectives, cultures, customs, values, and life experiences. Student expectations are shaped by their families and traditions added to their own lived experience and aspirations. Most students are working to support themselves or their families, and others have family caregiving responsibilities. Their satisfaction with the curriculum can be influenced by all of these factors and the roles and demands that they juggle. Some students are explicitly directed by their family's wishes, while other students persist in spite of grim family circumstances to achieve baccalaureate degrees. It is important to recognize that student satisfaction measures can be influenced by any number of things including performance expectations or even how well-course schedules accommodate their commutes and work schedules.

It is not uncommon for students to dwell on future employment prospects more than the educational process at hand. Particularly for students who do not come from privileged backgrounds, remunerable employment is central to their motivation to attend college. Although education is a vehicle for obtaining knowledge and skills that can be applied in job settings, academic institutions set different priorities than employment agencies. Entry level job opportunities in public health are not always obvious to students. To prepare students to search for jobs and to present themselves in the job market, students need additional guidance and reassurance. Because these skills are important but non-academic in nature, they are presented through a mandatory, no-credit professional topics seminar and individual coaching through the Career Services Office in Student Affairs. Students find encouragement in the stories of credible informants, namely recent graduates who have found satisfying jobs. For a proportion of undergraduate students, the focus is on the next leg of their educational journeys. They, too, want to hear from students who have successfully matriculated into the graduate and professional programs to which they aspire.

### Institutional Change

#### Education as a Business and Financial Performance of Programs

Most campus budgets are driven by tuition revenue, and few institutions these days are brimming with reserves. Since undergraduate education enrollments are the largest proportion of tuition income, there is constant pressure to grow enrollments to support more costly graduate programs and other institutional needs and priorities. Cohort models have historically been associated with high impact but lower enrollment (2). Resnick et al. (49) review of undergraduate public health programs confirms the wide variability of programs from curriculum to enrollment, even among the subset of programs accredited by CEPH (49). Like the proliferation of many other health related programs, baccalaureate programs in public health have been viewed as revenue producing opportunities and as such are subject to being regarded as a means to an end. Consequently, curriculum decisions may be driven by economics rather than best practices.

A notable academic who, in his frustration to respond to the constant demand for institutional and accreditation metrics, coined the phrase, “the tyranny of metrics” (50). While his commentary pertained largely to measurement of student outcomes and faculty productivity, the tyrannical metrics can easily be expanded to include enrollment targets, tuition revenue data, retention rates, and graduation statistics. Education incurs costs, but the value of that education must also be considered. Acknowledging that educational institutions (and departments and programs) need to stay afloat, a purely business/accounting approach to educational endeavors can seem antithetical to the values we aim to engender through public health education. That is to say, the dollars, income and expenditures, don't tell the whole story. Some public health programs have boasted of hundreds of students enrolled in their programs or hundreds of graduates annually, and new programs continue to proliferate with the hope of the sacred cash cow arriving to ameliorate whatever financial wows a campus might be experiencing. This, however, does not seem to be a very mindful approach to designing meaningful education nor to developing communities of conscience. Employing high impact practices in education and especially implementing the learning community/cohort approach may not yield the profit margins of less intentional and more convenient and economical knowledge transfer approaches, but lasting value…now that's another matter that raises Smith's question of purpose (48).

#### The Challenge of Purpose

Reflecting on the purpose of the academic institution might well be the greatest determinant of the choices made about curricular approaches and pedagogies. The options are circumscribed by the priorities, so just what are the priorities? If an academic institution is genuinely devoted to best practices and maximal educational benefit, then strategies like learning communities and cohort models are viable choices. The question then becomes, *how* can we implement these approaches given the resources at hand? If, on the other hand, a more utilitarian view of education prevails, some good disperse across the greatest number, then the focus shifts to the economics of education, e.g., how can limited resources be distributed to be of some benefit to the greatest numbers of students? Depending on the philosophical positions and values of an institution, a college, or a department, different options will be considered. Invoking the old adage, *we value what we measure* may be appropriate to these considerations. If the metrics we choose are limited to enrollments, graduation statistics, and job placement figures, we risk bypassing and missing the impacts of learning communities and cohort models that are not so easily reduced. Somehow, we must reconcile the economics with curricular integrity.

#### Clarifying the Purpose of Undergraduate Public Health Education

The Institute of Medicine Report (10) that stimulated the rapid growth of undergraduate public health programs asserts that the point of education is to yield an educated citizenry. That is a broad educational objective that has been infused into many different endeavors some of which may not be compatible with very intentional curriculum, learning communities, and cohort models. Nevertheless, learning communities are valuable for solidifying values and mirroring collaboration in the public health arena affording students opportunities to fail and succeed in a learning environment while preparing for the ambiguities of the real world. At this point in the evolution of undergraduate programs, the landscape is just coming into focus (49). Some programs represent very intentional curriculum intended to prepare students to enter the public health work force. Others offer foundational public health degrees via a menu of public health courses culminating in baccalaureate degrees that serve as pipelines to other health related career trajectories (e.g., MD, DDS, DVM). Students in pre-health science programs will likely form primary professional identities outside public health even if they are informed by public health understandings. These different ends are likely to invoke different means, and cohort models and intentional learning communities may not be compatible with all undergraduate initiatives. Especially for programs intentionally preparing graduates to fulfill entry level roles in the public health workforce, learning communities remain a highly relevant educational strategy.

## Questions for Further Consideration

As the undergraduate public health movement evolves, the full picture will become clearer. As has occurred within other disciplines and academic programs, there will likely be some adjustment in both the number and the nature of programs. Among the questions to be answered are:

- Which educational models are most compatible and effective in public health education?
- Is there a critical level of best practices needed in public health education? How much is enough (dosage) to yield the desired effect?
- How is undergraduate education in general and programs in specific impacting the public health workforce?
- Which educational approaches and strategies satisfy institutional Cost Effectiveness/Cost-Benefit analyses?
- In what ways are pedagogical choices limited by revenue expectations in undergraduate programming?

To answer the questions, it is imperative that we re-examine and affirm the philosophical commitments of our academic institutions and of public health education. We must take into account our public health values and ethical commitments remembering that we are preparing students to address complex problems of vulnerable populations. The AACU endorsed best educational practices are likely to endure in any analysis, but we are challenged to find better ways to measure the extent of their impact. Given that the power and impact of learning communities/cohort models continue to resurface, it is likely that this strategy will hold a key to meaningful education and “deep learning” in undergraduate public health education.

## Author Contributions

KO conceptualized and wrote this article reflecting the collaboratively developed undergraduate program at the University of Illinois, School of Public Health.

### Conflict of Interest Statement

The author declares that the research was conducted in the absence of any commercial or financial relationships that could be construed as a potential conflict of interest.

## Footnotes

^1^The University of Illinois at Chicago (UIC) is Chicago's only public research university, and it includes 30,000 students, 15 colleges, a hospital and a health sciences system. It is one of three campuses in the University of Illinois system.

^2^The diversity of the UIC student body has been recognized by many agencies and sources over the last decade. Current diversity information can be found at, https://www.hr.uic.edu/diversity_at_uic/.

^3^Current institutional data can be found at, https://oir.uic.edu/data/student-data/

^4^Students can petition to replace PUBH393 Fieldwork in Public Health with another selective course, in which case they will complete 12 core courses for a total of 32 semester hours.

^5^*Selectives* refers to courses that are part of a specific menu of courses from which students chose.

^6^The curriculum is in full compliance with the CEPH undergraduate learner outcomes. Due to the integrated rather than discipline-specific nature of the curriculum, CEPH competencies for baccalaureate level education tend to map to multiple rather than singular courses.

^7^The *Framing the Future* initiative was launched in 2014 and spearheaded by ASPPH to re-examine public health education.

^8^UIC School of Public Health is accredited by the Council for Education in Public Health through 2022.

^9^The matrix model is predicated on the work of Stake, Stufflebeam, and Quinn-Patton. It entails goal directed program evaluation and accommodates both qualitative and quantitative indicators reflecting the intersection of planning, implementation, and outcomes relative to faculty, content, and students. Evaluation priorities change and the model is designed to capture data and information pertaining to the questions raised.

^10^*Race to the Top* was a $4 billion fund launched by the federal government in 2009 to incentivize states and school districts to innovate and improve education and student outcomes.

^11^No Child Left Behind was a 2002 reauthorization of the Elementary and Secondary Education Act enacted by Congress to “close student achievement gaps by providing all children with a fair, equal, and significant opportunity to obtain a high-quality education.”

^12^Current institutional data, including graduation rates, can be found at, https://oir.uic.edu/data/student-data/.

^13^The major GPA for graduation will be adjusted to 2.5 in AY2019/20, which may impact future graduation rates.

^14^A very high proportion of students on campus are PELL eligible, reported as 57% in AY2017/18.
